# The role of routine SARS-CoV-2 screening of healthcare-workers in acute care hospitals in 2020: a systematic review and meta-analysis

**DOI:** 10.1186/s12879-022-07554-5

**Published:** 2022-07-02

**Authors:** J. M. Jabs, A. Schwabe, A. D. Wollkopf, B. Gebel, J. Stadelmaier, S. Erdmann, F. Radicke, H. Grundmann, A. Kramer, I. Monsef, G. Rücker, J. Rupp, S. Scheithauer, C. Schmucker, A. Simon, Nico T. Mutters

**Affiliations:** 1 Institute for Hygiene and Public Health, Bonn University Hospital, Venusberg-Campus 1, 53127 Bonn, Germany; 2 Department of Infectious Diseases and Microbiology, University Hospital Schleswig-Holstein, Campus Lübeck, Ratzeburger Allee 160, 23538 Lübeck, Germany; 3 Institute for Evidence in Medicine, Faculty of Medicine and Medical Center, University of Freiburg, Breisacher Str. 86, 79110 Freiburg, Germany; 4 Institute for Hygiene and Environmental Medicine, University Medicine Greifswald, Ferdinand-Sauerbruch-Straße, 17475 Greifswald, Germany; 5 Institute for Infection Prevention and Hospital Hygiene, Faculty of Medicine and Medical Center, University of Freiburg, Hugstetter Straße 55, 79106 Freiburg, Germany; 6 Department I of Internal Medicine, Center for Integrated Oncology Aachen Bonn Cologne Duesseldorf, Cochrane Haematology, University Hospital of Cologne, Kerpener Str. 62, 50937 Cologne, Germany; 7 Institute of Medical Biometry and Statistics, Faculty of Medicine and Medical Center, University of Freiburg, Zinkmattenstraße 6a, 79108 Freiburg, Germany; 8 Institute of Infection Control and Infectious Diseases, University Medical Center Göttingen, Robert-Koch-Straße 40, 37075 Göttingen, Germany; 9 Clinic for Pediatric Oncology and Hematology, Saarland University Hospital, Kirrberger Straße, 66421 Homburg, Saar Germany

**Keywords:** COVID-19, SARS-CoV-2, Coronavirus, Screening, Healthcare-workers, Infection control, Prevention, PCR, Hospital

## Abstract

**Background:**

Healthcare workers (HCW) are at increased risk of infection with SARS-CoV-2. Vulnerable patient populations in particular must be protected, and clinics should not become transmission hotspots to avoid delaying medical treatments independent of COVID. Because asymptomatic transmission has been described, routine screening of asymptomatic HCW would potentially be able to interrupt chains of infection through early detection.

**Methods:**

A systematic search was conducted in the Cochrane COVID-19 Study Register, Web of Science and WHO COVID‐19 Global literature on coronavirus with regard to non-incident related testing of healthcare workers using polymerase chain reaction on May 4th 2021. Studies since January 2020 were included. An assessment of risk of bias and representativeness was performed.

**Results:**

The search identified 39 studies with heterogeneous designs. Data collection of the included studies took place from January to August 2020. The studies were conducted worldwide and the sample size of the included HCW ranged from 70 to 9449 participants. In total, 1000 of 51,700 (1.9%) asymptomatic HCW were tested positive for SARS-CoV-2 using PCR testing. The proportion of positive test results ranged between 0 and 14.3%. No study reported on HCW-screening related reductions in infected person-days.

**Discussion and conclusions:**

The heterogeneous proportions might be explained by different regional incidences, lock-downs, and pre-analytical pitfalls that reduce the sensitivity of the nasopharyngeal swab. The very high prevalence in some studies indicates that screening HCW for SARS-CoV-2 may be important particularly in geographical regions and pandemic periods with a high-incidence. With low numbers and an increasing rate of vaccinated HCW, a strict cost–benefit consideration must be made, especially in times of low incidences. Since we found no studies that reported on HCW-screening related reductions in infected person-days, re-evaluation should be done when these are available.

**Supplementary Information:**

The online version contains supplementary material available at 10.1186/s12879-022-07554-5.

## Introduction

To control the global SARS-CoV-2 pandemic, measures such as personal protective equipment (PPE), disinfection, virucidal gargling and nasal spray [1], window ventilation or mechanical ventilation systems, public restrictions such as business closures, contact and visitor restrictions, vaccination etc. are being used. The long-term effects of these measures, especially on social life and the economic situation are difficult to assess. HCW have an increased risk of infection due to their exposure and occupational intensity of contacts [2]. The possibility of asymptomatic infection in HCW increases the risk of nosocomial transmission to "non-COVID" patients and to other HCW [3]. Nosocomial infection or even unprotected exposure of HCW necessitates interruptions in their availability and aggravates any pre-existing shortage of HCW in specialised inpatient services. In addition, HCW might suffer from associated fears of infection, isolation, and transmission to their own families [4]. Ultimately, material shortages of PPE in the past meant that staff safety could not be guaranteed at all times. Nosocomial infections, which account for approximately 20% of patient and 89% of HCW infections with SARS-CoV-2 in the United Kingdom [5, 6], have been described as sometimes even having a more severe and complex course [7]. Therefore, many hospitals screen patients on admission, regardless of contacts or symptoms, while HCW are tested only when symptomatic. But the disease may present with minimal or no symptoms [8] and asymptomatic transmission has been described in up to 50% of cases [9]. Nosocomial infections account for 12–29% of these [10]. Similar numbers and durations of viral infection were observed as in symptomatic individuals [11, 12]. Considering these risks, regular routine screening of HCW would be a conceivable tool to control the pandemic as it may protect the hospital staff themselves and, in particular, the vulnerable patient populations from transmission by HCW [7].

Additionally HCW morale and mental health have been boosted by screening programs in past pandemics [13]. Hospitals have special roles in pandemics, as patients with serious comorbidities or new-onset diseases sometimes delay seeking medical treatment in fear of infection with SARS-CoV-2, which may worsen their prognosis [14]. Limitations to extend screening programs by also considering asymptomatic HCW include financial as well as capacity and logistical problems, and the risk of massive workforce losses if a considerable number of HCW are tested positive, sometimes also false-positive [15]. Thus, appropriate screening programs must be well considered and planned. We conducted a systematic review to summarise the existing literature on routine SARS-CoV-2 screening of HCW in acute care hospitals using PCR to demonstrate the usefulness of screening for HCW.

## Methods

### Systematic literature search

This systematic review is reported according to the PRISMA (Preferred Reporting Items for Systematic Reviews and Meta-Analyses) 2020 guideline [16].

For the identification of studies systematic literature searches were performed by an information specialist and peer reviewed by a second information specialist.

On May 4th 2021 we searched for studies that screened for SARS-CoV-2 with PCR in HCW. The following sources were searched: the Cochrane COVID-19 Study Register (comprising MEDLINE, Embase, CENTRAL ClinicalTrials.gov, WHO ICTRP, medRxiv, RetractionWatch), Web of Science (Science Citation Index Expanded and Emerging Sources Citation Index) and WHO COVID‐19 Global Global literature on coronavirus. The search term included different variants of HCW, SARS-CoV-2 and PCR. The detailed search strategies are available as additional material (Additional file 1).

Five reviewers conducted a title and abstract screening. In a second step reports potentially meeting the inclusion criteria were read in full-text to finally decide for inclusion.

### Inclusion and exclusion criteria

Inclusion criteria were (i) any HCW of any age and gender, without symptoms working in hospitals settings, (ii) non-cause-related screening for SARS-CoV-2 conducted by reverse transcriptase polymerase chain reaction (RT-PCR) testing (additional rapid test/serology was possible/allowed).

Cause-related testing was not excluded per se, but recorded separately, although this was not explicitly sought. The same applies to studies reporting on screening programmes in nursing homes or homecare services, which are also described, but not included for further analysis.

Outcomes considered were (i) reduction of infected person-days of HCW, (ii) and/or number of positive tested HCW (overall, asymptomatic).

Included study types were (i) randomized controlled trials (RCTs), (ii) non-RCTs (including quasi RCTs using inappropriate strategies of randomly allocating interventions), cross-sectional studies, cohort studies, controlled before-and-after studies, interrupted time series and (iii) any type of evidence synthesis (e.g., systematic reviews) if primary data were available or for identifying relevant additional studies.

Exclusion criteria were (i) testing of non-medical staff, (ii) performance of exclusively rapid tests / serology, (iii) exclusively cause-related screening (contacts, symptoms) for SARS-CoV-2 and (iv) any type of modelling studies.

### Data extraction

The following data were extracted independently by the reviewers: (i) key study characteristics (bibliographical data, study design, geographical area where data were collected, period of data collection, mean age, gender and number of included HCW); (ii) Number tested, number positive tested asymptomatic, Reduction of infected person days; (iii) Setting [level 1: Primary Care (Primary Care Physician, Family Physician or Public Health Clinic); level 2: Specialty Physician Care (Specialist Physician); level 3: Hospital Care (Acute Care General Hospital or Ambulatory Surgical Center); level 4: Specialty Hospital Care (Specialty Acute Care Hospital], ward (ICU, emergency, regular); (iiii) relevant exclusion criteria.

Missing results were reported, but not included in further analysis.

### Data analyses

For the meta-analysis, the R package meta (Version 4.18-0) was used [17, 18]. Proportions were calculated with exact binomial 95% confidence intervals (CI) and visualized using a forest plot, including a 95%-prediction interval to depict the range of proportions across the available and potential future studies. Higgins’ I^2^ was used to describe the estimated proportion of variability due to heterogeneity between studies rather than random error [19]. If appropriate, proportions were pooled using a random intercept logistic regression model [20].

### Risk of bias and representativeness

The risk of bias and the representativeness of the results was assessed considering pre-defined criteria which were developed by our group based on other epidemiological research [21]. Thereby, risk of bias assessment was based on the completeness of data, i.e., whether all recruited HCW (whole study sample) were considered when data were analysed (low risk of bias) or whether data were missing (e.g. due to drop-outs; high risk of bias). Data representativeness based on the characteristics of the study sample; i.e., when a selected sample (e.g. HCW from a high-risk region) was considered to derive estimates, representativeness was judged as “low”, whereas data representativeness was judged as “high” when the study included a broad-ranging sample reflecting HCW worldwide.

Of note, for both data extraction and the methodological assessments, we relied on information provided in the individual study reports. If no judgment could be made owing to missing information (poor reporting), the corresponding item for risk of bias or data representativeness was classified as “unclear”.

## Results

### Study selection process

**Fig. 1:**
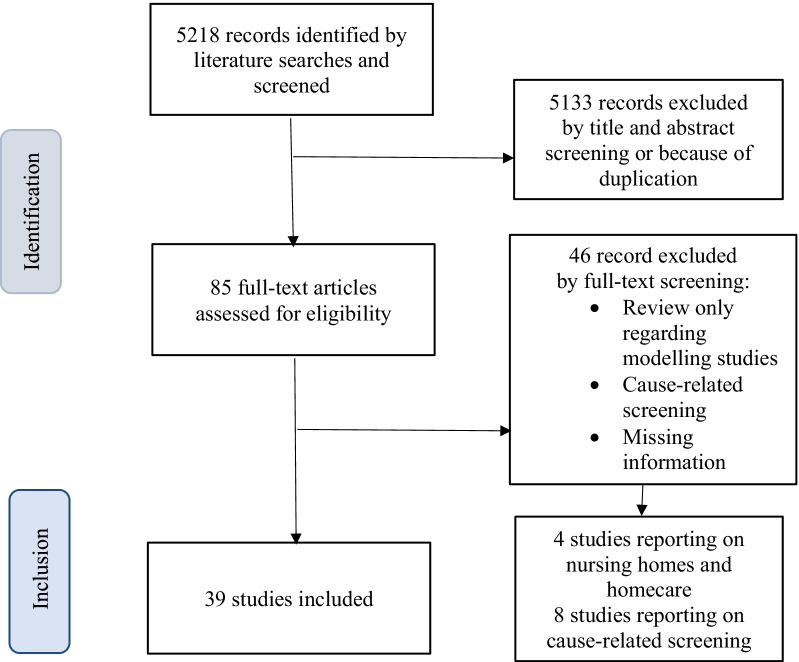
Flowchart of study selection process (PRISMA-flowchart)

Figure 1 (PRISMA flowchart) presents the study selection process in detail [22].

The searches yielded 5218 records, of which 39 studies including 51,700 HCW met the inclusion criteria (reporting on non-cause-related screening of HCW).

In addition, we found eight studies that reported on cause-related testing, including 7.950 samples of HCW, which is described separately.

### Study characteristics

Table 1 presents the details of the 39 included studies.

**Table 1:** Study characteristics and results of included studies

First author	Study type	Country	Population description	No. pos tested asymptomatic/ sample size	Setting (level)	Ward	Period of data collection	Mean age of HCW	Gender distribution (female in %)
Abdelmoniem et al. [23]	Cross-sectional	Egypt	HCW (nurses, physicians, patient transporters, cleaners, radiologists, administrative staff)	29/203 (14.3%)	3	3	01.–14.06.2020	31.9	49
Al-Zoubi et al. [24]	Cohort	Jordan	HCW (nurses, physicians, other staff)	0/370 (0%)	4	0	18.03.–29.04.2020	32.02	33
Armin et al. [25]	Cross-sectional	Iran	HCW, office workers, hospital service workers	25/475 (5.3%)	3	0	20.04. 05.05.2020	N. A	80
Brown et al. [26]	Cross-sectional	UK	HCW of six hospitals (medical and non-medical staff)	23/1152 (2.0%)	0	0	24.04.–07.05.2020	39 (median)	70
Campbell et al. [27]	Cohort	USA	HCW	16/525 (3%)	3	0	N. A	N. A	N. A
Cavicchiolo et al. [28]	Cohort	Italy	HCW	3/112 (2.7%)	4	1	21.02.–21.04.2020	N. A	N. A
Demmer et al. [29]	Cohort	USA	HCW	0/488 (0%)	3–4	6	20.04.–24.06.2020	41	84.2
Dillner et al. [30]	Cohort	Sweden	HCW	235/9449 (11.8%)	4	6	23.04.–24.06.2020	N. A	79.3
Fakhim et al. [31]	Cross-sectional	Iran	HCW	14/102 (13.7%)	3–4	0	20.02.–15.03.2020	N. A	67.6
Favara et al. [32]	Cohort	UK	Patient-facing HCW (nurses, doctors, other patient-facing staff)	0/70 (0%)	3	2	01.–07.06.2020	42	56.6
Ferreira et al. [33]	Cross-sectional	Canada	HCW (nurses, physicians, allied health professionals)	Cohort 1: 9/1669 (0.54%) Cohort 2: 20/4107 (0.49%)	3	0	17.04.–29.05.2020	N. A	N. A
Fusco et al. [34]	Cross-sectional	Italy	HCW (nurses, physicians, other staff)	2/115 (1.7%)	4	5	23.03.–02.04.2020	43	48.7
Guery et al. [35]	Cross-sectional	France	HCW	3/136 (2.2)	4	2	16.–19.04.2020	39 (median)	82
Halbrook et al. [36]	Cohort	USA	frontline HCW and first responders of County Fire Department	10/1787 (0.6%) of all 4/1108 (0.4%) of HCW	4	0	08.04.–31.08.2020	N. A	64
Handal et al. [37]	Cross-sectional	Norway	HCW	12/360 (3.3%)	4	4	11.05.–11.06.2020	N. A	76.4
Hellewell et al. [38]	Cohort	UK	HCW	15/200 (7.5%)	4	0	26.03.–05.05.2020	N. A	N. A
Hidayat et al. [39]	Cross-sectional	Indonesia	HCW and other staff from an university hospital	83/742 (11.1%)	4	6	19. -23.06.2020	N. A	66.9
Horton et al. [40]	Cross-sectional	USA	HCW	4/5826 (0.09%)	4	0	22.04.–02.06.2020	N. A	N. A
Huang et al. [41]	Cross-sectional	USA	HCW (clinical staff, administrative staff, food services workers, environmental services)	0/1394 (0%)	4	0	01.04.–15.06.2020	N. A	N. A
Jameson et al. [42]	Cohort	USA	HCW (respiratory therapists, providers, nurses, patient care assistants)	0/121 (0%)	3–4	6	N. A	N. A	N. A
Johnson et al. [43]	Cohort	USA	HCW from four hospitals	1/439 (0.2%)	3–4	6	21.05.–16.07.2020	N. A	N. A
Kantele et al. [44]	Cross-sectional	Finland	HCW	36/1095 (3.3%)	4	6	22.04.2020	38 (median)	82.7
Kassem et al. [45]	Cross-sectional	Egypt	HCW at gastroenterological service	9/74 (12.2%)	4	2	01.–14.04.2020		59.5
Lahner et al. [46]	Cross-sectional	Italy	Health Workers	58/2057 (2.7%)	3	0	18.03.–27.04.2020	45.2	60.2
Lai et al. [47]	Case series	China	HCW	3/335 (0.9)	4	6	01.01.–09.02.2020		73.6
Lombardi et al. [48]	Cross-sectional	Italy	HCW	41/1093(3.7%)	4	6	24.02.–31.03.2020	44.5	64.2
Martin et al. [49]	Cross-sectional	Belgium	HCW	31/270(11.5%)	4	4	N. A	37	73
Mohanty et al. [50]	Cross-sectional	USA	HCW and patients	64/1670 (3.8%) in total; 33/912 HCW	0	0	02.04.–30.06.2020	42.5	48.6
Moncunill et al. [51]	Cohort	Spain	HCW	25/501 (5.0%)	3–4	6	27.04. 06.05.2020	42	71.7
Moolla et al. [52]	Cohort	South Africa	HCW (nurses, administrative staff, doctors, general assistants)	12/799(8.3%)	0	0	01.05.–31.05.2020	39.7	77.4
Olalla et al. [53]	Cross-sectional	Spain	HCW (doctors, nurses, nursing assistants, security guards, administrative and cleaning staff)	2/498 (0.4%)	3	6	15.–25.04.2020	41.5	80
Olmos et al. [54]	Cross-sectional	Chile	HCW	14/414 (3.4%)	3	6	01.05.–01.07.2020	33	76
Oster et al. [55]	Cohort	Israel	HCW (medical, nursing, paramedical, administrative staff)	5/4897 (0.1%)	4	6	23.03.–11.05.2020	N. A	N. A
Rivett et al. [15]	Cross-sectional	UK	HCW	31/1032 (3%)	4	6	06.–24.04.2020	34	71
Stock et al. [56]	Cross-sectional	USA	HCW (adult clinician)	8/98 (8.2%)	4	6	04.–20.04.2020	37.6	50
Temkin [57]	Cross-sectional	Israel	HCW	1/522 (0.2%)	3	4	30.04.–07.05.2020	39.33	63.98
Treibel et al. [58]	Cohort	UK	HCW (doctors, nurses, allied health professionals, administrators, and others)	53/1479 (3.6%)	3	0	23.–31.03.2020	N. A	N. A
Vahidy et al. [59]	Cross-sectional	USA	HCW (Covid-Facing, Non Covid-Facing, Non-Clinical)	112/2787 (4%)	3–4	0	N. A	40.68	73
Zhou et al. [12]	Cross-sectional	China	HCW (doctors, nurses, administrative staff, clinical support staff)	28/3674 (0.76%)	4	0	16.–25.03.2020	N. A	67.7

*HCW* healthcare worker, *N.A* not applicable, Level 1: Primary Care (Primary Care Physician, Family Physician or Public Health Clinic); Level 2: Specialty Physician Care (Specialist Physician); Level 3: Hospital Care (Acute Care General Hospital or Ambulatory Surgical Center); Level 4: Specialty Hospital Care (Specialty Acute Care Hospital)

In short, data collection took place between January 2020 [47] and August 2020 [36] and sample sizes of PCR tested HCW ranged from 70 [32] to 9449 samples [30]. The studies were conducted in all six WHO defined regions (Africa, America, South-East Asia, Europe, Eastern Mediterranean and Western Pacific), most of the samples were taken in the USA (27,385 samples).

17 studies (n = 12,229) reported on mean age of HCW. Mean age ranged from 31.9 years [23] to 45.2 years [46] with an overall mean age of 40.6 years. 29 studies (n = 30,931) reported on gender distribution of tested HCW. Proportion of women ranged from 33 [24] to 84.2% [29], resulting in an overall proportion of 71.7%. Tested participants included doctors, nurses, allied health professionals, emergency first responders, healthcare assistants, physiotherapists, administrators, security guards, cleaning staff, food service workers and patient transporters. These were working in ICU, Emergency ward and Regular ward, 17 studies did not further report on the corresponding wards. 24 of the included studies used a cross-sectional design, 15 studies were based on cohorts (prospective cohort studies without control groups) and one study was a case series. RT-PCR testing was used in all studies. A total of 36 studies were conducted at acute care hospitals, three studies did not provide any information regarding the facilities’ level.

The studies on nursing homes, home care services and additional studies on cause-related testing are described in Tables 2 and 3, with no relevant differences in study characteristics compared to non-cause-related testing.

**Table 2:** Study characteristics and results of studies on nursing homes

First Author	Study type	Country	Population description	No. pos tested asymptomatic/sample size	Setting (Level)	Ward	Period of data collection	Mean Age of HCW	Gender distribution (female in %)
Bayle et al. [60]	Cohort	France	All asymptomatic or pauci-symptomatic nursing home employees	32/241 (13.3%)	8	8	16.–29.04.2020	39.9	83.8
Hassan et al. [61]	Cohort	Sweden	Employees of five home care service companies	13/387 (3.3%)	8	9	11.05.–17.06.2020	43	52.6
McBee et al. [62]	Cross-sectional	USA	Staff and residents of 123 nursing homes	31/13687 (0.2%) and 35/1,639 (2.1%)	8	0	21.04.–08.05.2020	N. A	N. A
Van Buul et al. [63]	Cross-sectional	Netherlands HCW	HCW	1/542 (0.002%)	8	0	04.–10.05.2020	45.7	91.3

*HCW*  healthcare worker, *N.A*  not applicable, Level 1: Primary Care (Primary Care Physician, Family Physician or Public Health Clinic); Level 2: Specialty Physician Care (Specialist Physician); Level 3: Hospital Care (Acute Care General Hospital or Ambulatory Surgical Center); Level 4: Specialty Hospital Care (Specialty Acute Care Hospital)

**Table 3:** Study characteristics and results of studies on cause-relating tests

First Author	Study type	Country	Population description	No. pos. tested asymptomatic/ sample size	Setting (Level)	Ward	Period of data collection	Mean Age of HCW	Gender distribution (female in %)
Borras-Bermejo et al. [64]	Cohort study	Spain	HCW and residents	n = 2655 Staff: 403/2655 tested positive for COVID-19 144/403 (55.8%) of staff members tested positive were asymptomatic	Nursing homes	Previous laboratory-confirmed cases of COVID-19	10.04.–24.04.2020	N. A	N. A
Harada et al. [65]	Cross-sectional design	Japan	HCW and patients	52/697 (7.5%)	Level 3	N. A	24.03.–24.04.2020	N. A	N. A
Khalil et al. [66]	Cohort study	UK	HCW	47/266 (18%), 16/47 (34%) were asymptomatic	Level 3	N. A	17.03.–16.04.2020	N. A	N. A
Rajme-López et al. [67]	Cross-sectional design	Mexico	HCW	111/2000 (5.5%)	N.A	N. A	28.04.–08.07.2020	34	57.5/42.5%
Rasmussen et al. [68]	Cohort study	Denmark	HCW	7/347 (1.9%)	Level 4	N. A	27.05.–03.06.2020	N. A	N. A
Sebastian et al. [69]	Cross-sectional design	Argentina	HCW	8/204 (4%)	Dental hospital	N. A	03/–10/2020	38	64/36%
Soltani-Zangbar et al. [70]	Cross-sectional design	Iran	HCW	66/609 (10.8%)	Level 3	N. A	04/–06/2020	41.9	38.75/61.25%
Zhao et al. [71]	Retrospective cohort study	China	HCW	88/1172 (9.7%) of HCW with close contact to confirmed cases of COVID-19	Level 4	N. A	14.01.–21.02.2020	N. A	N. A

*HCW*  healthcare worker, *N.A*  not applicable, Level 1: Primary Care (Primary Care Physician, Family Physician or Public Health Clinic); Level 2: Specialty Physician Care (Specialist Physician); Level 3: Hospital Care (Acute Care General Hospital or Ambulatory Surgical Center); Level 4: Specialty Hospital Care (Specialty Acute Care Hospital)

### Outcomes reported

**Fig. 2:**
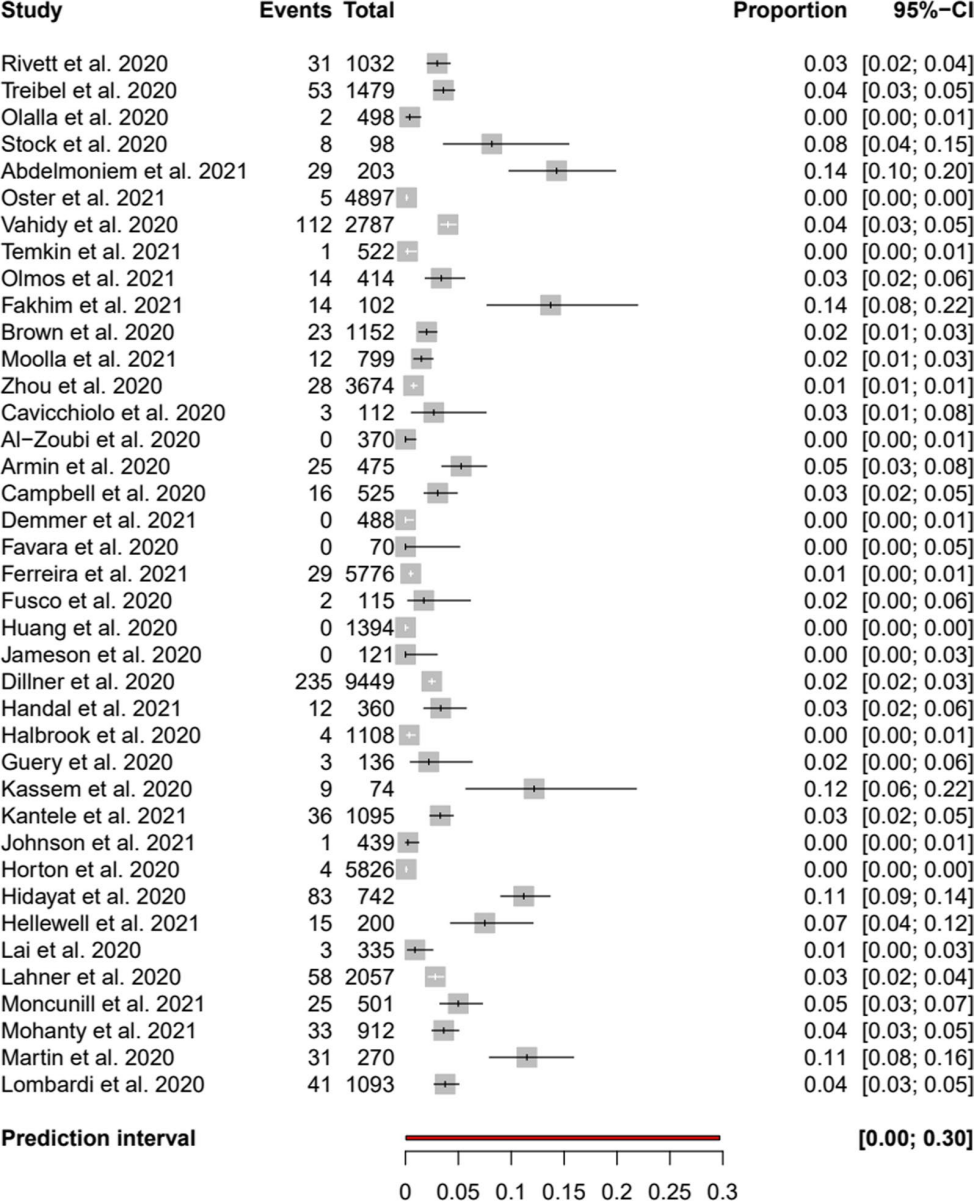
Forest plot of proportion of positive test results in asymptomatic healthcare workers

In total 1000 (1.9%) of 51,700 HCW were tested positive. Figure 2 presents a forest plot of the positive rate of asymptomatically tested HCW. We abstained from presenting a pooled estimate and a confidence interval because of the large between-study heterogeneity (I^2^ = 94.5% with 95% CI 93.3–95.5%).

Thereby, the proportion of positive test results of screened HCW ranged from 0.0% [24, 29, 32, 41, 42] to 14.3% [23] (Table 1). None of the studies reported infected person-days or reduction of these.

In the non-systematically considered studies reporting on cause-related testing of HCW, 782 of 7950 samples were positive, with the proportion of positive test results ranging from 1.9 to 34%. The four studies on screening of asymptomatic HCW in nursing homes and home care services reported on 77 positive test results in 14,857 tested individuals (0.5% in total, ranging from 0.002 to 13.3%).

### Assessment of risk of bias and representativeness

The results of the respective assessments are shown in Table 4.

**Table 4:** Assessment of risk of bias (RoB) and representativeness of included studies

Study	Risk of bias Are date for the full sample available and used for estimation of prevalence?	Representativeness Is the data representative for HCW worldwide?
Abdelmoniem et al. [23]	Low RoB	Lacking representativeness
Al-Zoubi et al. [24]	Low RoB	Lacking representativeness
Armin et al. [25]	Low RoB	Lacking representativeness
Brown et al. [26]	Low RoB	Potentially for hospital staff, wide range of diverse job roles
Campbell et al. [27]	Low RoB	Lacking representativeness
Cavicchiolo et al. [28]	Low RoB	Lacking representativeness
Demmer et al. [29]	Low RoB	Lacking representativeness
Dillner et al. [30]	Low RoB	Potentially for hospital staff, high case number
Fakhim et al. [31]	Low RoB	Lacking representativeness
Favara et al. [32]	Low RoB	Lacking representativeness
Ferreira et al. [33]	Low RoB	Potentially for hospital staff, high case number
Fusco et al. [34]	Low RoB	Lacking representativeness
Guery et al. [35]	Low RoB	Lacking representativeness
Halbrook et al. [36]	Low RoB	Potentially for staff of hospitals and fire departments, high case number
Handal et al. [37]	Low RoB	Lacking representativeness
Hellewell et al. [38]	Low RoB	Lacking representativeness
Hidayat et al. [39]	Low RoB	Lacking representativeness
Horton et al. [40]	Low RoB	Lacking representativeness
Huang et al. [41]	Low RoB	Lacking representativeness
Jameson et al. [42]	Low RoB	Lacking representativeness
Johnson et al. [43]	Low RoB	Lacking representativeness
Kantele et al. [44]	Low RoB	Potentially for hospital staff, high case number, different risks of C-19 contact
Kassem et al. [45]	Low RoB	Lacking representativeness
Lahner et al. [46]	Low RoB	Lacking representativeness
Lai et al. [47]	Low RoB	Lacking representativeness
Lombardi et al. [48]	Low RoB	Potentially for nursing homes, high case number
Martin et al. [49]	Low RoB	Potentially for nursing homes, high case number
Mohanty et al. [50]	Low RoB	Lacking representativeness
Moncunill et al. [51]	Low RoB	Lacking representativeness
Moolla et al. [52]	Low RoB	Lacking representativeness
Olalla et al. [53]	Low RoB	Lacking representativeness
Olmos et al. [54]	Low RoB	Lacking representativeness
Oster et al. [55]	Low RoB	Lacking representativeness
Rivett et al. [15]	Low RoB	Lacking representativeness
Stock et al. [56]	Low RoB	Lacking representativeness
Temkin [57]	Low RoB	Lacking representativeness
Treibel et al. [58]	Low RoB	Lacking representativeness
Vahidy et al. [59]	Low RoB	Lacking representativeness
Zhou et al. [12]	Low RoB	Potentially for hospital staff, wide range of diverse job roles

*HCW*  healthcare worker

## Discussion

This systematic review aimed to summarise the existing literature on routine SARS-CoV-2 screening of HCW in acute care hospitals. We identified 39 studies, which took place from January to August 2020 (first and second wave of the pandemic). A total of 1000 (1.9%) of 51,700 asymptomatic HCW tested positive for SARS-CoV-2. Individuals were positive in up to 14.3% of the tested individuals [23], the lowest detection rate was 0% [24, 29, 32, 41, 42].

The data on routine testing of HCW are heterogeneous and ambiguous, as the forest plot (Fig. 2) demonstrates. No underlying cause could be found, therefore pooling or subgroup analysis was not suitable. The varying numbers might be explained by regional differences in incidences and/or baseline features of the pandemic in the different countries. The SARS-CoV-2 pandemic has exhibited a substantial diachronous habit and therefore baseline features as well as measures such as lock-downs [15, 72], or in general surveillance efforts might have inflated or conversely deflated local incidence rates. The included studies collected their data from January 2020 during the first COVID-19 wave, until August 2020, hence effects of vaccination will not yet have impacted the results.

In general, higher positive rates among asymptomatic HCW can be expected if incidence increases in the overall population due to a higher probability of exposure to SARS-CoV-2 positive close contacts outside the hospital setting. This was confirmed by the study of Shields et al. in which the parallel determination of SARS-CoV-2 immunglobulin-G showed high rates of expired infections, contrasting very low detection rates of positives in RT-PCR [73]. But in the context of low circulation of the virus screening of asymptomatic HCW was poorly effective in the identification of virus-spreading HCW [74]. On the opposite, the highest proportion of asymptomatic patients is detectable in Egypt, which could be seen as representative for countries with younger demographic structures and a high incidence in the population [23]. In cases of such immensely high detection rates, early detection may be able to prevent a relevant proportion of transmissions, especially if high incidences are associated with a low hygiene adherence. In high-prevalence regions and situations, screening of asymptomatic HCW could therefore be a useful and recommendable additional measure to established prevention strategies. A modelling study concluded that weekly screening of asymptomatic staff in an emergency department could reduce new HCW and patient infections by 5.1% within 30 days (Assuming a constant 1.2 new infections per 10,000 persons) and by 21.1% within 30 days at higher incidences (Assuming a constant 3.7 new infections per 10,000 persons) [75]. The associated risk of transmission to vulnerable patient groups by HCW as well as the more severe course described for nosocomial transmissions should also be considered. While the stringent use of PPE not only protects the HCW but also close contact patients, this barrier is not unbreachable since in clinical practice adherence to the complex prevention bundle is not expected to reach 100% [76].

Regarding risk of bias assessment RT-PCR as an objective method and gold standard for the diagnosis of SARS-CoV-2 was used as an assessment tool of infection in all studies. Nevertheless, preanalytics, which can significantly reduce sensitivity of the test, must be considered [77]. These were not reported in detail in particular, neither transport routes nor the qualifications of the samplers were listed. Testing scenarios in level 3 and 4 facilities were predominant, thus limiting data representativeness of the entire global population and facilities of other levels, especially level 1 (primary care) and level 2 (specialist physician).

Additionally, we non-systematically found studies reporting on cause-related testing of HCW, showing higher detection rates (9.8% vs. 1.9%). Due to higher pre-test probability, those numbers are not surprising. However, given the fact our initial search for relevant literature did not focus on this population, our results lack representativeness. The same applies to our results on HCW in nursing homes and home care providers, showing a lower proportion of positive tested compared to HCW working in hospitals (0.5% vs. 1.9%).

At the time the included studies took place, no vaccine was yet available for widespread use.

Currently, the majority of HCW in developed countries are vaccinated against SARS-CoV-2. However, the benefits of screening regimens among asymptomatically vaccinated individuals are even more unclear due to the lower and shorter infectivity [78], but possibly an inverse effect through an increased feeling of safety, and lower prevalence of COVID-19 among vaccinated individuals [79]. Emerging variants of SARS-CoV-2 like Omicron with possibly reduced vaccine effectiveness [80], as well as the continued development of vaccines and test methods could influence the usefulness of those prevention and control strategies in the near future. Rapid PCR tests [81] and PCR mass tests [82] have been developed, but cannot be used on a regular and widespread base yet, because they require a high logistical effort.

At the time the systematic review was conducted, there was no evidence screening for HCW can lead to reduced transmission rates. However, asymptomatic SARS-CoV-2 carriers can lead to transmission [83, 84]. Thus, it is plausible that screening in vulnerable areas may subsequently lead to a reduction in infected person days. If unscreened asymptomatic SARS-CoV-2 positive HCW continue working, transmission to patients and staff could occur, resulting in relevant staff absences that may compromise medical care. The current state of evidence, however, does not firmly support unconditional HCW screening. From a public health perspective screening asymptomatic HCW e.g. several times a week is a costly exercise with unknown effect on transmission rates, in particular since standard infection control measures such as wearing medical masks—namely surgical masks or FFP2/KN95/N95 masks—were commonly implemented in hospital settings worldwide during the pandemic (Additional file 1).

In total, asymptomatic SARS-CoV-2 infections were detected in a relatively small proportion of HCW; accordingly, in times of low incidence strict trade-offs must be made in terms of feasibility and cost-effectiveness. Unfortunately, we did not find trials evaluating endpoints such as reduction in nosocomial infected person-days. In addition, up until completion of this review, no planned or ongoing trials with this outcome were registered at clinicaltrials.gov.

Currently there are two ongoing studies registered on clinicaltrials.gov investigating how COVID-19 spreads among HCW (ClinicalTrials.gov Identifier: NCT04574765, NCT04370119). We are looking forward to the results of these studies.

## Conclusions

Asymptomatic infections of HCW and a possible associated risk of transmission to vulnerable patient populations may impact patient safety. Additionally, reducing nosocomial transmission between HCW is important in pandemic control since staff absences impact healthcare for all patients negatively and in particular SARS-CoV-2 patients needing mechanical ventilation. Our findings indicate that asymptomatic infections in HCW vary widely. Screening HCW for SARS-CoV-2 at regular intervals thus seems reasonable in times and regions of higher incidence. However, no certain incidence level can currently be determined for starting routine screening in a cost-effective way. Clinical studies investigating the reduction of infected person-days by routine screening are currently lacking. In particular since new variants of SARS-CoV-2 will continue to appear that might change transmission dynamics, implementing surveillance in critical structures such as the healthcare sector seems nevertheless appropriate.

## Supplementary Information

**Additional file 1:** Search term.

## Data Availability

All the data generated and/or analysed are included in this published article and its additional information files.

## Abbreviations

HCW: Healthcare worker
RT-PCR: Reverse transcriptase polymerase chain reaction
PPE: Personal protective equipment
WHO: World Health Organisation
RCT: Randomized controlled trial
ICU: Intensive care unit
