# Supplementary material for: The role of routine SARS-CoV-2 screening of healthcare-workers in acute care hospitals in 2020: a systematic review and meta-analysis
Source: BMC Infect Dis. 2022 Jul 2;22:587. doi: 10.1186/s12879-022-07554-5 (PMC9250183; doi:10.1186/s12879-022-07554-5)
Supplement: Supplementary file 1 — Additional file 1: Search term. [file 12879_2022_7554_MOESM1_ESM.docx]

**Additional material**

**Cochrane COVID-19 Study Register (https://covid-10.cochrane.org)**

(includes: Medline, Embase, CENTRAL, ClinicalTrials.gov, WHO ICTRP, medRxiv, RetractionWatch)

Searchstring:

"polymerase chain reaction" or PCR* or "RT-qPCR" or "RT-qPCRs" or "RT-PCR" or "RT-PCRs" or "rRT-PCR" or "rCT-PCRs" OR screen* OR surveillance* OR "diagnostic accuracy"

AND

HCW or "health team" or "emergency worker" or "emergency workers" or midwife* or midwives or physiotherapist* or physician* or clinician* or "health personnel" or "health practitioner" or "health practitioners" or "health worker" or "health workers" or "health provider" or "health providers" or "health staff" or "health professional" or "health professionals" or "healthcare personnel" or "healthcare practitioner" or "healthcare practitioners" or "healthcare worker" or "healthcare workers" or "healthcare provider" or "healthcare providers" or "healthcare staff" or "healthcare professional" or "healthcare professionals" or "care personnel" or "care practitioner" or "care practitioners" or "care worker" or "care workers" or "care provider" or "care providers" or "care staff" or "care professional" or "care professionals" or "hospital worker" or "hospital workers" or "hospital staff" or "hospital personnel" or "hospital administrator" or "hospital administrators" or "hospital transmission" or "hospital transmissions" or "medical worker" or "medical workers" or "medical staff" or "medical professional" or "medical professionals" or "medical personnel" or "medical administrator" or "medical administrators" or "care unit worker" or "care unit workers" or "care unit staff" or "care unit professional" or "care unit professionals" or "care unit personnel" or "icu worker" or "icu workers" or "icu staff" or "icu professional" or "icu professionals" or "icu personnel" or nurs* or physician* or doctor* or paramedic* or "emergency medical technician" or "emergency medical technicians" or "emergency medical assistant" or "emergency medical assistants" or "emergency department staff" or "medical student" or "medical students" or anesthesist* or anaesthesist* or anesthesiologist* or anaesthesiologist* or cardiologist* or dentist* or "dental practitioner" or "dental practitioners" or "dental staff" or "dental assistant" or "dental assistants" or "general practitioner" or "general practitioners" or geriatrician* or internist* or neurosurgeon* or nephrologist* or neurologist* or ophthalmologist* or neurologist* or pediatrician* or paediatrician* or "palliative care specialist" or "palliative care specialists" or "palliative care staff" or pharmacist* or psychiatrist* or pulmologist* or radiologist* or rheumatologist* or surgeon* or "surgical staff" or urologist*

Study characteristics:

1) "Intervention assignment": "Randomised" OR "Quasi-Randomised" OR "Non-Randomised" OR "Unclear"

2) "Study aim": "diagnostic/prognostic" OR "Health Services Research" OR "Transmission" AND "Study design": "Parallel/Crossover" OR "single arm/controlled before after" OR "case series/case control/cohort" OR "Time series" OR "Unclear" OR "Other" OR "cross-sectional"

**Web of Science (Core Collection)**

• Science Citation Index Expanded (1945-present)

• Emerging Sources Citation Index (2015-present)

• Limit to 2020 - 2021

#1

AB=(coronavir* OR coronovir* OR (coron* NEAR/2 (virus* OR viral* OR virinae*) ) OR COVID OR COVID19 OR ncov OR n-cov OR 2019nCoV OR nCoV2019 OR WN-CoV OR WNCoV OR HCoV-19 OR HCoV19 OR 2019 novel* OR 2019 nCoV OR 2019nCoV OR SARS-CoV-2 OR SARSCoV-2 OR SARSCoV2 OR SARS-CoV2 OR SARSCov19 OR SARS-CoV19 OR SARSCov-19 OR SARS-CoV-19 OR Ncovor OR Ncorona* OR Ncorono* OR NcovWuhan* OR NcovHubei* OR NcovChina* OR NcovChinese* OR SARS2 OR SARS-2) OR TI=(coronavir* OR coronovir* OR (coron* NEAR/2 (virus* OR viral* OR virinae*) ) OR COVID OR COVID19 OR ncov OR n-cov OR 2019nCoV OR nCoV2019 OR WN-CoV OR WNCoV OR HCoV-19 OR HCoV19 OR 2019 novel* OR 2019 nCoV OR 2019nCoV OR SARS-CoV-2 OR SARSCoV-2 OR SARSCoV2 OR SARS-CoV2 OR SARSCov19 OR SARS-CoV19 OR SARSCov-19 OR SARS-CoV-19 OR Ncovor OR Ncorona* OR Ncorono* OR NcovWuhan* OR NcovHubei* OR NcovChina* OR NcovChinese* OR SARS2 OR SARS-2)

#2

TI=(polymerase chain reaction OR PCR* OR RT-qPCR* OR RT-PCR* OR rRT-PCR* OR screen* OR surveillance* OR "diagnostic accuracy") OR AB=(polymerase chain reaction OR PCR* OR RT-qPCR* OR RT-PCR* OR rRT-PCR* OR screen* OR surveillance* OR "diagnostic accuracy")

#3

TI=((HCW OR health team* OR emergengcy worker* OR midwife* OR midwives* OR ((physical OR occupational) NEAR/2 therapist*) OR physiotherapist* OR (therapist* NEAR/2 hospital) OR physician* OR clinician*)) OR AB=((HCW OR health team* OR emergency worker* OR midwife* OR midwives* OR ((physical OR occupational) NEAR/2 therapist*) OR physiotherapist* OR (therapist* NEAR/2 hospital) OR physician* OR clinician*))

#4

TI=(nurs* OR physician* OR doctor* OR paramedic* OR emergency medical technician* OR emergency medical assistant* OR medical student* OR anesthesist* OR anaesthesist* OR anesthesiologist* OR anaesthesiologist* OR cardiologist* OR dentist* OR dental practitioner* OR dental staff OR dental assistant* OR general practitioner* OR geriatrician* OR internist* OR neurosurgeon* OR nephrologist* OR neurologist* OR ophthalmologist* OR neurologist* OR pediatrician* OR paediatrician* OR palliative care specialist* OR palliative care staff OR pharmacist* OR psychiatrist* OR pulmologist* OR radiologist* OR rheumatologist* OR surgeon* OR surgical staff OR urologist* OR hospital transmission*) OR AB=(nurs* OR physician* OR doctor* OR paramedic* OR emergency medical technician* OR emergency medical assistant* OR medical student* OR anesthesist* OR anaesthesist* OR anesthesiologist* OR anaesthesiologist* OR cardiologist* OR dentist* OR dental practitioner* OR dental staff OR dental assistant* OR general practitioner* OR geriatrician* OR internist* OR neurosurgeon* OR nephrologist* OR neurologist* OR ophthalmologist* OR neurologist* OR pediatrician* OR paediatrician* OR palliative care specialist* OR palliative care staff OR pharmacist* OR psychiatrist* OR pulmologist* OR radiologist* OR rheumatologist* OR surgeon* OR surgical staff OR urologist* OR hospital transmission*)

#5

TI=(((key OR front-line OR frontline OR emergency) NEAR/3 (staff OR worker* OR workforce OR manforce OR personnel OR practitioner* OR professional*) )) OR AB=(((key OR front-line OR frontline OR emergency) NEAR/3 (staff OR worker* OR workforce OR manforce OR personnel OR practitioner* OR professional*) ))

#6

TI=((icu NEAR/1 (worker* OR staff OR profession* OR personnel OR team*) )) OR AB=((icu NEAR/1 (worker* OR staff OR profession* OR personnel OR team*) ))

#7

TI=((health* NEAR/1 (worker* OR practitioner* OR professional* OR staff OR personnel OR employee* ) )) OR AB=((health* NEAR/1 (worker* OR practitioner* OR professional* OR staff OR personnel OR employee*) ))

#8

TI=((care unit* NEAR/1 (worker* OR staff OR profession* OR personnel) )) OR AB=((care unit* NEAR/1 (worker* OR staff OR profession* OR personnel) ))

#9

TI=(medical NEAR/1 (worker* OR staff OR profession* OR personnel OR administrator*) ) OR AB=(medical NEAR/1 (worker* OR staff OR profession* OR personnel OR administrator*) )

#10

AB=(((health-care OR healthcare OR hospital) NEAR/3 (worker* OR practitioner* OR professional* OR staff OR personnel OR employee*) )) OR TI=(((health-care OR healthcare OR hospital) NEAR/3 (worker* OR practitioner* OR professional* OR staff OR personnel OR employee*) ))

#11

#10 OR #9 OR #8 OR #7 OR #6 OR #5 OR #4 OR #3

#12

#11 AND #2 AND #1

#13

AB=(random* OR placebo OR trial OR groups OR "phase 3" or "phase3" or p3 or "pIII") OR TI=(random* OR placebo OR trial OR groups OR "phase 3" or "phase3" or p3 or "pIII")

#14

AB=(cohort OR (control AND study) OR (control AND group*) OR epidemiologic studies OR program OR comparative stud* OR evaluation studies OR follow-up* OR time factors OR cross-sectional*) OR TI=(cohort OR (control AND study) OR (control AND group*) OR epidemiologic studies OR program OR comparative stud* OR evaluation studies OR follow-up* OR time factors OR cross-sectional*)

#15

#12 AND (#13 OR #14)

**WHO COVID-19 database (search.bvsalud.org/global-literature-on-novel-coronavirus-2019-ncov/)**

HCW or "health team" or emergency worker* or "emergency workers" or midwife* or midwives or physiotherapist* or physician* or clinician* or "health personnel" or "health practitioner" or "health practitioners" or "health worker" or "health workers" or "health provider" or "health providers" or "health staff" or "health professional" or "health professionals" or "healthcare personnel" or "healthcare practitioner" or "healthcare practitioners" or "healthcare worker" or "healthcare workers" or "healthcare provider" or "healthcare providers" or "healthcare staff" or "healthcare professional" or "healthcare professionals" or "care personnel" or "care practitioner" or "care practitioners" or "care worker" or "care workers" or "care provider" or "care providers" or "care staff" or "care professional" or "care professionals" or "hospital worker" or "hospital workers" or "hospital staff" or "hospital personnel" or "hospital administrator" or "hospital administrators" or "hospital transmission" or "hospital transmissions" or "medical worker" or "medical workers" or "medical staff" or "medical professional" or "medical professionals" or "medical personnel" or "medical administrator" or "medical administrators" or "care unit worker" or "care unit workers" or "care unit staff" or "care unit professional" or "care unit professionals" or "care unit personnel" or "icu worker" or "icu workers" or "icu staff" or "icu professional" or "icu professionals" or "icu personnel" or nurs* or physician* or doctor* or paramedic* or "emergency medical technician" or "emergency medical technicians" or "emergency medical assistant" or "emergency medical assistants" or "emergency department staff" or "medical student" or "medical students" or anesthesist* or anaesthesist* or anesthesiologist* or anaesthesiologist* or cardiologist* or dentist* or "dental practitioner" or "dental practitioners" or "dental staff" or "dental assistant" or "dental assistants" or "general practitioner" or "general practitioners" or geriatrician* or internist* or neurosurgeon* or nephrologist* or neurologist* or ophthalmologist* or neurologist* or pediatrician* or paediatrician* or "palliative care specialist" or "palliative care specialists" or "palliative care staff" or pharmacist* or psychiatrist* or pulmologist* or radiologist* or rheumatologist* or surgeon* or "surgical staff" or urologist*

AND

"polymerase chain reaction" or PCR* or "RT-qPCR" or "RT-qPCRs" or "RT-PCR" or "RT-PCRs" or "rRT-PCR" or "rCT-PCRs" or screen* or surveillance* or "diagnostic accuracy"

AND

(random* or placebo or trial or groups or "phase 3" or "phase3" or p3 or "pIII" or cohort or (control and study) or (control and group*) or epidemiologic studies or program or comparative stud* or evaluation studies or follow-up* or time factors or cross-sectional*)

- Advanced search; search fields: title, abstract, subject
- without Medline, Pubmed and ICTRP
